# A Statistical Analysis of the Relationship between Harmonic Surprise and Preference in Popular Music

**DOI:** 10.3389/fnhum.2017.00263

**Published:** 2017-05-18

**Authors:** Scott A. Miles, David S. Rosen, Norberto M. Grzywacz

**Affiliations:** ^1^Interdisciplinary Program in Neuroscience, Georgetown UniversityWashington, DC, United States; ^2^Department of Neuroscience, Georgetown UniversityWashington, DC, United States; ^3^Applied Cognitive and Brain Sciences, Drexel UniversityPhiladelphia, PA, United States; ^4^Department of Physics, Georgetown UniversityWashington, DC, United States; ^5^Graduate School of Arts and Sciences, Georgetown UniversityWashington, DC, United States

**Keywords:** music cognition, music preference, information theory, expectation violation, neuroaesthetics, music perception, popular music

## Abstract

Studies have shown that some musical pieces may preferentially activate reward centers in the brain. Less is known, however, about the structural aspects of music that are associated with this activation. Based on the music cognition literature, we propose two hypotheses for why some musical pieces are preferred over others. The first, the Absolute-Surprise Hypothesis, states that unexpected events in music directly lead to pleasure. The second, the Contrastive-Surprise Hypothesis, proposes that the juxtaposition of unexpected events and subsequent expected events leads to an overall rewarding response. We tested these hypotheses within the framework of information theory, using the measure of “surprise.” This information-theoretic variable mathematically describes how improbable an event is given a known distribution. We performed a statistical investigation of surprise in the harmonic structure of songs within a representative corpus of Western popular music, namely, the McGill Billboard Project corpus. We found that chords of songs in the top quartile of the Billboard chart showed greater average surprise than those in the bottom quartile. We also found that the different sections within top-quartile songs varied more in their average surprise than the sections within bottom-quartile songs. The results of this study are consistent with both the Absolute- and Contrastive-Surprise Hypotheses. Although these hypotheses seem contradictory to one another, we cannot yet discard the possibility that both absolute and contrastive types of surprise play roles in the enjoyment of popular music. We call this possibility the Hybrid-Surprise Hypothesis. The results of this statistical investigation have implications for both music cognition and the human neural mechanisms of esthetic judgments.

## Introduction

Although music has been said to have no apparent direct survival value (Pinker, 1997), it can elicit pleasurable reward responses in the human brain like those associated with food or sex (Blood et al., 1999; Blood and Zatorre, 2001; Salimpoor et al., 2011, 2013). Recent neuroimaging work has revealed many of the mechanisms recruited by the brain during the perception of reward from listening to music that is preferred (Pereira et al., 2011; Salimpoor et al., 2013). Less is known, however, about what structural aspects of music are required to drive these mechanisms. A prevailing theory of this process states that adherence to or deviation from listeners' expectations leads to emotional reward (Meyer, 1956; Huron, 2006). One challenge in testing this theory of how music structure leads to reward is to quantify expectations. Another challenge is to test the theory in an ecologically valid way, that is, avoiding the artificial stimuli that are often used in laboratory tests, which can feature isolated chords (e.g., Koelsch et al., 2001) or a novel musical system (Loui and Wessel, 2008).

The framework of information theory has proved to be useful in quantifying expectations and deviations from them in naturalistic music. Through information theory, one can investigate expectations based on statistical measures of representative corpora. Such an approach was proposed by Meyer (1957), and has been expanded upon by other researchers in subsequent years (e.g., Knopoff and Hutchinson, 1981; Temperley, 2007; Agres et al., 2013; for a review, see Rohrmeier and Koelsch, 2012). While he was cautious about overstating how much statistical analysis could reveal, Meyer claimed that knowledge about deviation from expectations, examined through probability, could help reveal their relationship to emotion, a primary source of meaning in music (Meyer, 1957). So far, however, information-theory-driven corpus analyses have primarily been aimed at describing aspects of music, with less emphasis on investigating any relationship these aspects might have with how the music is perceived. We set out to extend such analyses to determine the capacity of aspects within popular music to evoke a reward response. In a first step toward achieving this goal, we investigate the relationship between two variables: one representing deviation from expectations, and another quantifying preference.

Within information theory, *surprise* is a mathematical measure of how much an event deviates from expectations (Atick, 1992). This measure has been used to quantify expectation violation in music (e.g., Egermann et al., 2013). In turn, the analysis of surprise in the harmonic structure of music in a large naturalistic corpus could be used as a measure of deviation from expectations. The McGill Billboard Project (Burgoyne et al., 2011) is such a corpus. The currently available dataset features transcriptions of 732 Western popular music songs chosen at random from the *Billboard Hot 100* charts over a 34-year period, extending from 1958 to 1991. The harmonic structure of the songs featured in the corpus should therefore be representative for popular music from that time period. The dataset provides information about chord types within the songs, allowing us to measure harmonic surprise, and use it as a variable to represent deviation from expectations.

The dataset also provides information about song *preference*, in as much as the peak chart position is reported for each song. During this period, chart positions were determined by two factors: record sales and radio airplay, with record sales being the overriding factor (Parker, 1991). Because chart positions are linked to record sales, songs with higher peak chart positions may be considered generally more highly preferred to those with lower chart positions. Thus, relationships between peak chart positions on the Billboard *Hot 100* could be used as a variable to represent preference.

With surprise and preference as operationalizable variables, the prevailing theory described above (Meyer, 1956; Huron, 2006) leads to two specific hypotheses on the relationship between deviation from expectations and reward value in music. The first hypothesis states that moderate increases in the absolute level of surprise raise pleasurable emotions, thus driving preference upward. We call this the *Absolute-Surprise Hypothesis*. The second hypothesis states that overall preference is driven by passages with moderately high surprise, thus evoking unpleasant emotions of “tension,” followed by passages with low surprise, evoking pleasurable “release” from these emotions. We call this the *Contrastive-Surprise Hypothesis*.

The Absolute-Surprise Hypothesis is premised on surprise itself being a good thing for the observer. Surprise indicates new information (Meyer, 1957), thus possibly being valuable to the person receiving it. As calculated in the present study, surprise is also known as *information content* (MacKay, 2003). Since dopamine has been associated with novelty-seeking behavior (Suhara et al., 2001), it is possible that the processing of harmonically surprising sections of music is associated with dopamine release, and therefore with reward response. In a study involving both functional magnetic resonance imaging (fMRI) and positron emission topography (PET), dopamine release was found to be simultaneous with the presentation of sections of music known to evoke “chills,” which often feature harmonically unexpected events (Salimpoor et al., 2011).

The Contrastive-Surprise Hypothesis, on the other hand, is premised on surprise being bad for the listener. This hypothesis is compatible with David Huron's concept of contrastive valence, which attributes one type of a listener's enjoyment of music to a release from the tension induced by surprise (Huron, 2006). This idea is also voiced by Meyer (1956, 1957), who allows for an overall “determinate meaning” drawn from the relationship between antecedents and consequences in a piece of music. In the music cognition literature, the nature of how such “antecedents” are perceived is currently much clearer than any role of the “consequences.” While we are not aware of any direct neuroscientific evidence of any contrastive-surprise effect on music preference, there is extensive work supporting the idea of harmonic surprise being unpleasant in music. Studies using electroencephalography (Patel et al., 1998; Koelsch et al., 2001), magnetoencephalography (Maess et al., 2001) and functional magnetic resonance imaging (Tillmann et al., 2003) have shown that harmonically unexpected events in music are processed by the brain similarly to syntactic errors in language.

Many of these and other music cognition studies examining harmonic expectation have used a series of bare, static chords, rather than presenting naturalistic music (e.g., Koelsch et al., 2001; Tillmann et al., 2003, but see Steinbeis et al., 2005, 2006). Other studies have used naturalistic stimuli to examine preference, but present evidence of neural mechanisms without addressing the structure of the music that might have driven those mechanisms (Pereira et al., 2011; Salimpoor et al., 2013). Interestingly, evidence from a behavioral study (Schellenberg et al., 2012) shows that contrast in the emotional character of alternatingly presented pieces of naturalistic music can be associated with increased preference.

In the present study, we test these two hypotheses through a statistical analysis of harmonic structure in Western popular music. We compare a set of highly preferred songs (songs in the top quartile of the Billboard corpus by peak chart position) with a set of less preferred songs (songs in the bottom quartile of the corpus). We perform our comparisons both at the level of entire songs and at the level of song sections. In doing so, we aim to determine how harmonic surprise might contribute to music preference.

The goal of this statistical analysis is to learn more about how the brain processes music, by examining the structure of music that is preferred. A similar statistical approach has been used to study the neuroscience of the visual system. The principle behind this approach is that one can often explain the mechanisms of a brain sensory system as optimized processors for ecologically important stimuli. Thus, statistical analyses examining natural images (Field, 1987; Ruderman and Bialek, 1994; Balboa and Grzywacz, 2003) led to a greater understanding of the visual system of the brain. For example, computational analyses have shown that the shapes of receptive fields in both the retina and the visual cortex are optimal for the extraction of information from such images (Field, 1987; Atick and Redlich, 1992; Balboa and Grzywacz, 2000). More recently, this approach has been adapted to the neuroesthetics of vision (Graham and Field, 2007; Graham and Redies, 2010). For example, Aleem et al. (2017) recently examined the role of complexity in visual neuroesthetics. To achieve this goal, they performed a statistical analysis of painted portraits. Similarly, the present study examines the statistical properties of harmonic surprise, a form of complexity in music. A greater understanding of the structural properties of preferable music could further elucidate neural reward mechanisms that drive music preference.

## Materials and methods

### Music analyzed

We ran several statistical analyses on the publicly available dataset associated with the McGill Billboard Project corpus (Burgoyne et al., 2011). The dataset in the Project featured chord-by-chord transcriptions of the harmony from 732 unique songs. The songs were selected to be representative of the distribution of songs featured on the weekly *Billboard Hot 100* charts between 1958 and 1991. The distribution of songs was representative in terms of year of release and chart position. To compare the harmonic relationships among the songs, we transposed all songs to a common key (C major). We excluded songs determined to be in a minor key and those featuring within-song modulations, to examine a single uniform measure of surprise. Songs were determined to be in a minor key if the number of chords with the tonic as their root and a minor third was greater than the number of chords with the tonic as their root and a major third. The result was that we examined 545 songs in our analyses.

We separated the corpus into quartiles, based on the peak Billboard chart position of each song. The difference in Billboard quartiles was used as a proxy measure of preference within popular music. The top quartile (Q1) represented widely preferred songs and the bottom quartile (Q4) represented less widely preferred songs. To determine Q1 and Q4, we first ranked songs by their peak chart position, using the Fractional Ranking method to take ties into account. Songs with ranking below 136.25 (a quarter of 545) and above 408.75 (three quarters of 545) were assigned to Q1 and Q4 respectively. Consequently, Q1 and Q4 were only approximate quartiles. There were 151 songs in Q1 and 150 songs in Q4.

**Figure 1:**
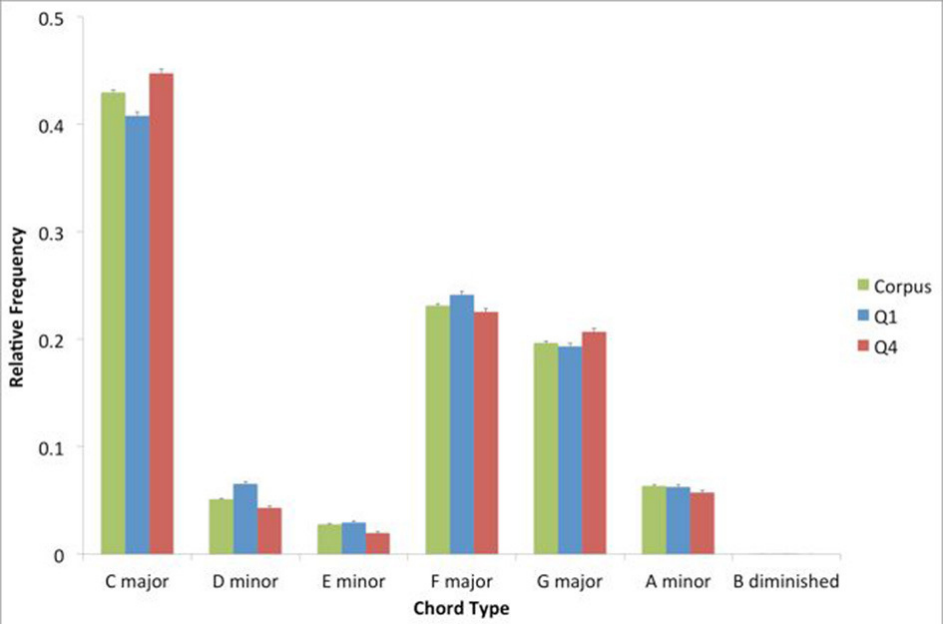
**Relative prevalence of each of the seven diatonic chords, within the entire corpus, top quartile (Q1) and bottom quartile (Q4)**. Error bars are standard errors. The prevalence of these chords is similar in the corpus, Q1, and Q4.

To determine the pattern of chord prevalence for the corpus, we looked at the distribution of chord types. We found 596 unique identifiers for chords. We then examined the distribution of the seven diatonic chords, after summing the frequency of all chords with common roots and thirds, regardless of extensions at the level of the fifth of the chord and beyond (Figure 1). We isolated these seven chord types only for the purposes of this specific analysis. In the analyses reported below, all 596 different types of chords were taken into consideration.

As Figure 1 shows, the patterns of prevalence for the corpus, Q1, and Q4 are similar. This pattern involves a hierarchy of prevalence where the I chord (C major in this case) is followed by the V and IV (G major and F major respectively). The pattern is consistent with the expected hierarchy of prevalence in Western tonal music (Berry, 1976; Krumhansl, 1990). Interestingly, there is a trend for the subdominant to be more prevalent than the dominant. This trend is a characteristic of the “rock” genre (Temperley, 2011), and is consistent with the hierarchy of prevalence reported in an analysis of another popular-music corpus (De Clercq and Temperley, 2011).

The duration of chords was not coded for these analyses. In the corpus transcription, a chord label was provided at the downbeat (first beat) of each measure, and additional chord labels were provided if a chord changed within the measure. We only included chord labels provided in the transcription in our statistical analyses of the corpus.

### Statistical measures

Let *N* be the number of different chords appearing in the corpus (*N* = 596 in this study). Let M_*j*_ be the number of times that Chord C_*j*_ (1 ≤ *j* ≤ *N*) appears. Thus, the total number of chords in the corpus, including repetitions, is $\sum_{i\;=\;1}^{N}M_{i}$. The probability that a chord picked at random from the corpus is C_*j*_ is

$$P\left(C_{j}\right)=\frac{M_{j}}{\sum_{i\;=\;1}^{N}M_{i}}\;,$$

from which, we calculate the surprise of finding C_*j*_ as

(1) $$S\left(C_{j}\right)=\;-log_{2}\left(P\left(C_{j}\right)\right).$$

In the paper, we report measurements from different types of statistics based on S(C_j_). In particular, we perform measurements across whole songs, individual sections, or transitions between consecutive sections. We begin by defining the notations for these quantities. We denote the r^th^ song of the q^th^ quartile by σ_q,r_, where 1 ≤ q ≤ 4 and N_q_ is the number of songs in the q^th^ quartile (151 in Q1 and 150 in Q4). Next, we denote the s^th^ section of Song σ by ψ_σ,*s*_, where N_σ_ is the number of sections in Song σ. Finally, we sometimes impose conditions on the chords contributing to a calculation. We label these conditions with the letter κ. For example, κ may be “entire song” or “a time interval within the song.”

The first measurement that we make uses (Equation 1) to obtain the average of surprise for either the entire song or a time interval within the song, i.e.,

(2) $${\bar{S}}_{q}\left(\kappa \right)=\frac{1}{N_{q}}\;\sum_{r=1}^{N_{q}}\sum_{j=1}^{N}P\left(C_{j}|\sigma_{q,r},\kappa \right)S\left(C_{j}\right).$$

Thus, we first fix the quartile and then average the surprises in each of its songs (the summation with Index j). We then take the mean across songs (the summation with Index r). The division by N_q_ is equivalent to the inclusion of P(σ_q,r_) in the summation performed to obtain this mean. We calculate it as in Equation (2) to discount the differential effects on expected surprise of songs that have different durations.

The second measurent that we make is an estimate of how much each Chord C_j_ contributes to the difference between Q1 and Q4. From Equation (2), the overall difference is

(3) $$\begin{array}{l} \Delta {\bar{S}}_{1,4}\text{​}\left(\kappa \right)={\bar{S}}_{1}\left(\kappa \right)-{\bar{S}}_{4}\text{​}\left(\kappa \right),\\ \Delta {\bar{S}}_{1,4}\text{​}\left(\kappa \right)=\;\frac{1}{N_{1}}\;\sum_{r=1}^{N_{1}}\sum_{j=1}^{N}P\left(C_{j}|\sigma_{1,r},\kappa \right)S\text{​}\left(C_{j}\right)\\ \;-\;\frac{1}{N_{4}}\sum_{r=1}^{N_{4}}\sum_{j=1}^{N}P\left(C_{j}|\sigma_{4,r},\kappa \right)S\left(C_{j}\right).\; \end{array}$$

If we group the two sums, we can decompose this summation to obtain the linear (additive) contribution of each Chord C_j_ as

(4) $$\begin{array}{l} \Delta {\bar{S}}_{1,4}\text{​}\left(C_{j},\kappa \right)=\\ \;\;S\left(C_{j}\right)\text{​}\left(\frac{1}{N_{1}}\;\sum_{r=1}^{N_{1}}P\left(C_{j}|\sigma_{1,r},\kappa \right)-\frac{1}{N_{4}}\;\sum_{r=1}^{N_{4}}P\left(C_{j}|\sigma_{4,r},\kappa \right)\right). \end{array}$$

To understand why this measurement represents the linear contribution of each chord to the difference in surprise between Q1 and Q4, perform the sum of Equation (4) over all chords (i.e., for 1 ≤ j ≤ N). This summation yields (Equation 3). We can interpret (Equation 4) as follows: the more surprising a chord is in the overall corpus, the larger possible effect this chord can have on the difference between Q1 and Q4. However, to have a large effect, the chord has to be much more prevalent on Q1 than on Q4. Importantly, a chord can have positive and negative contributions to the difference. Although S(C_j_) is always positive, the term inside the large parentheses in the right hand side can be negative. We would get negative contributions for chords that are more prevalent in Q4 than Q1. The negativity expresses that the chord contributes more to the mean surprise in the bottom quartile than in the top one.

The final measurements that we make in this paper capture properties of surprise for individual sections of songs. We begin by measuring the average surprise for section *s* of song *r* in quartile *q* as follows:

(5) $${\bar{S}}_{q,r}\left(s,\kappa \right)=\sum_{j=1}^{N}P\left(C_{j}|\psi_{\sigma_{q,r},s},\kappa \right)S\left(C_{j}\right).$$

Thus, we first fix the quartile (q), song (r), and section (s), and then obtain the average of surprises in it. From Equation (5), we calculate the first statistic related to sections as

$$\begin{array}{l} {\bar{S}}_{q,r,1}\text{​}\left(\kappa \right)=\frac{1}{N_{\sigma_{q,r}}}\sum_{s=1}^{N_{\sigma_{q,r}}}{\bar{S}}_{q,r}\left(s,\kappa \right),\\ {\bar{S}}_{q,r,2}\text{​}\left(\kappa \right)=\frac{1}{N_{\sigma_{q,r}}}\sum_{s=1}^{N_{\sigma_{q,r}}}{\bar{S}}_{q,r}^{2}\left(s,\kappa \right), \end{array}$$

and

(6) $$\varsigma_{q}\text{​}\left(\kappa \right)=\frac{1}{N_{q}}\underset{r=1}{\sum^{N_{q}}}\sqrt{{\bar{S}}_{q,r,1}^{2}\left(\kappa \right)-{\bar{S}}_{q,r,2}\left(\kappa \right)}.$$

Thus, to compute ς_q_, we first estimate the standard deviation of the average surprises of sections across every song in a quartile (the square-root term in Equation 6). We then calculate the mean of these standard deviations across songs. This mean is the summation with Index r divided by N_q_. Having calculated ς_q_ thus gives an indication of the variability of surprise across sections and hence, an indication of contrastive surprise.

Finally, we are interested in the difference in average surprise in the transition between consecutive sections. In particular, we have an interest in verses followed by choruses. To detect and use these verses, we define the functions

$$\phi \left(\psi_{\sigma ,s}\right)=\{\begin{array}{cc} 1 & if\;\psi_{\sigma ,s}\;is\;a\;verse\;\;\&\;\;\psi_{\sigma ,s+1}\;is\;a\;chorus\\ 0 & otherwise \end{array}.$$

and

$$\Delta {\bar{S}}_{q,r}\text{​}\left(s,\kappa \right)={\bar{S}}_{q,r}\text{​}\left(s,\kappa \right)-{\bar{S}}_{q,r}\text{​}\left(s+1,\kappa \right).$$

With these functions and (Equation 5), we define the final three measurements in this paper as

(7) $$\delta_{q,||}\text{​}\left(\kappa \right)=\frac{\sum_{r=1}^{N_{q}}\sum_{s=1}^{N_{\sigma_{q,r}}-1}\phi \left(\psi_{\sigma_{q,r},s}\right)\left|\;\Delta {\bar{S}}_{q,r}\left(s,\kappa \right)\right|}{\sum_{r=1}^{N_{q}}\sum_{s=1}^{N_{\sigma_{q,r}}-1}\phi \left(\psi_{\sigma_{q,r},s}\right)},$$

(8) $$\begin{array}{l} \delta_{q,-}\left(\kappa \right)=\\ \;\frac{\sum_{r=1}^{N_{q}}\sum_{s=1}^{N_{\sigma_{q,r}}-1}\phi \left(\psi_{\sigma_{q,r},s}\right)\left(\Delta {\bar{S}}_{q,r}\left(s,\kappa \right)-\left|\;\Delta {\bar{S}}_{q,r}\left(s,\kappa \right)\right|\right)/2}{\sum_{r=1}^{N_{q}}\sum_{s=1}^{N_{\sigma_{q,r}}-1}\phi \left(\psi_{\sigma_{q,r},s}\right)\frac{\left(\Delta {\bar{S}}_{q,r}\left(s,\kappa \right)-\left|\;\Delta {\bar{S}}_{q,r}\left(s,\kappa \right)\right|\right)}{2\Delta {\bar{S}}_{q,r}\left(s,\kappa \right)}}, \end{array}$$

and

(9) $$\begin{array}{l} \delta_{q,+}\text{​}\left(\kappa \right)=\\ \frac{\sum_{r=1}^{N_{q}}\sum_{s=1}^{N_{\sigma_{q,r}}-1}\phi \left(\psi_{\sigma_{q,r},s}\right)\left(\Delta {\bar{S}}_{q,r}\left(s,\kappa \right)+\left|\;\Delta {\bar{S}}_{q,r}\left(s,\kappa \right)\right|\right)/2}{\sum_{r=1}^{N_{q}}\sum_{s=1}^{N_{\sigma_{q,r}}-1}\phi \left(\psi_{\sigma_{q,r},s}\right)\frac{\left(\Delta {\bar{S}}_{q,r}\left(s,\kappa \right)+\left|\;\Delta {\bar{S}}_{q,r}\left(s,\kappa \right)\right|\right)}{2\Delta {\bar{S}}_{q,r}\left(s,\kappa \right)}}. \end{array}$$

Equation (7) gives the mean of the absolute values of the difference in surprise between verses and following choruses. This equation achieves this goal by only counting verse-to-chorus transitions through the Function Φ. Equation (8) gives the mean of the difference in surprise between verses and following choruses for cases in which the difference is negative. This result is obtained by manipulating the terms after the Function Φ in both the numerator and denominator. In the numerator, the term is equal to the difference when it is negative, but is zero otherwise. In turn, the term in the denominator is 1 when the difference is negative, but is zero otherwise. Equation (9) does the same as Equation (8), but for cases in which the difference is positive.

## Results

### The effect of absolute surprise

**Figure 2:**
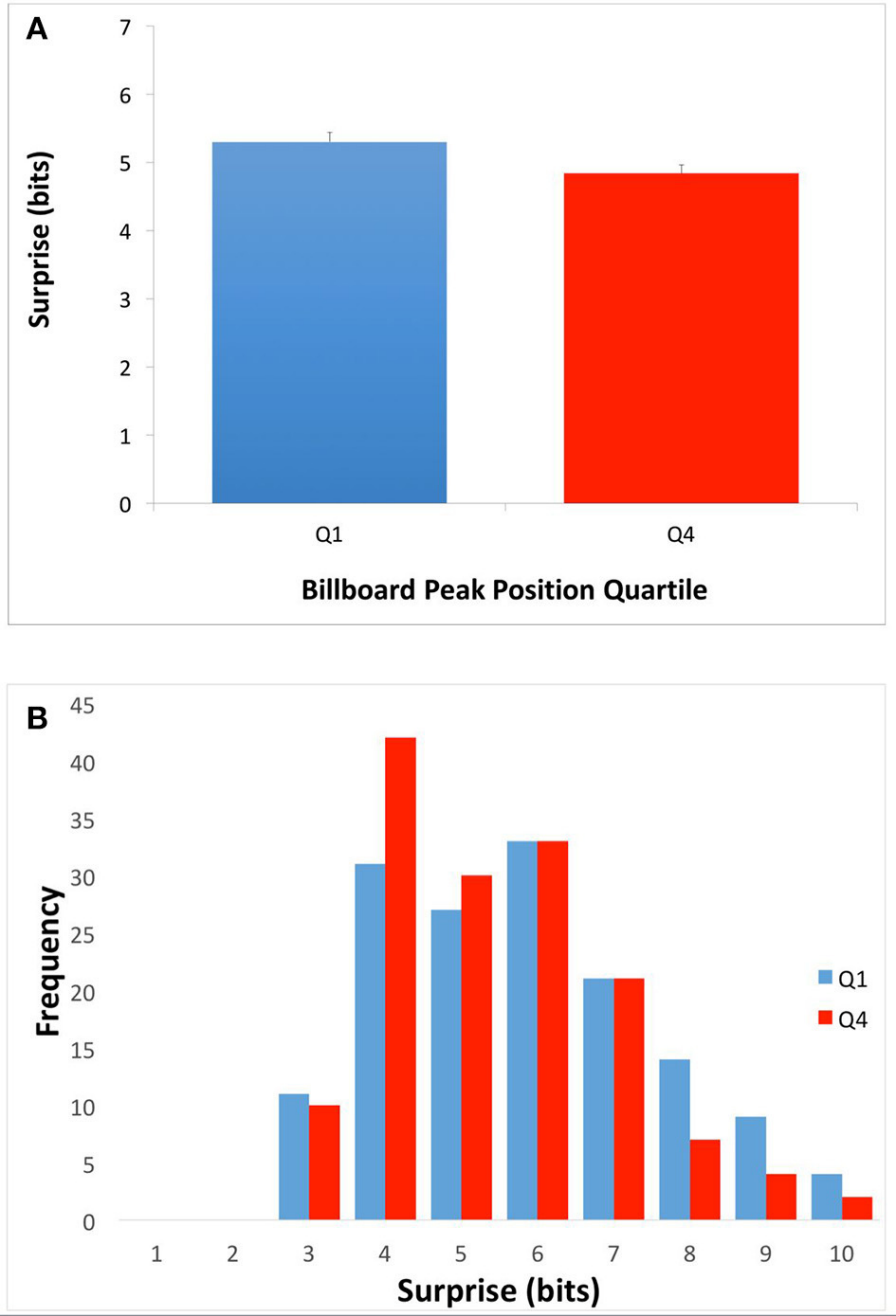
**Average song surprise for top-quartile (Q1) and bottom-quartile (Q4) songs**. **(A)** Mean and standard errors of surprise (Equation 2 with κ set to “whole song.”). **(B)** Frequency of average song surprise for Q1 and Q4. Q1 songs had higher mean surprise than Q4 songs. This superiority of Q1 songs is consistent with the Absolute-Surprise Hypothesis.

We set out to test two hypotheses for how the harmonic structure of popular music might drive pleasurable reward responses in the brain. The first, the Absolute-Surprise Hypothesis, states that moderate increases in the absolute level of surprise directly raise pleasurable emotions, thus driving music preference upward. To test this hypothesis, we examined the mean surprise of songs in top (Q1) and bottom (Q4) quartiles of the McGill Billboard Project corpus. To calculate this mean surprise, we used Equation (2) with κ set to “whole song.” If the Absolute-surprise Hypothesis was right, we would expect to see higher mean surprise in Q1 songs than in Q4 songs. The results of the comparison between Q1 and Q4 appear in Figure 2.

As Figure 2A shows, Q1 songs had significantly higher overall mean surprise than Q4 songs. The difference was small but statistically significant (Q1 mean = 5.31 bits, Q4 mean = 4.84 bits; two-tailed *t*-test; *t* = 2.48; *df* = 299; *p* < 0.01; Cohen's *d* = 0.289). To get more details of the difference, we plotted the histogram of chord surprises (Equation 1) for Q1 and Q4 (Figures 2B,C respectively). The histograms show considerable overlap, however while the histogram for Q1 songs showed a peak at around 6-bits surprise, the one for Q4 songs showed a peak at 4 bits. In addition, the Q1 histogram had a longer tail into larger surprises. Therefore, the Absolute-surprise Hypothesis is supported by our data, providing evidence that moderate increases in the absolute level of surprise of a song may indeed drive music preference upward.

### Time course of absolute-surprise effect

**Figure 3:**
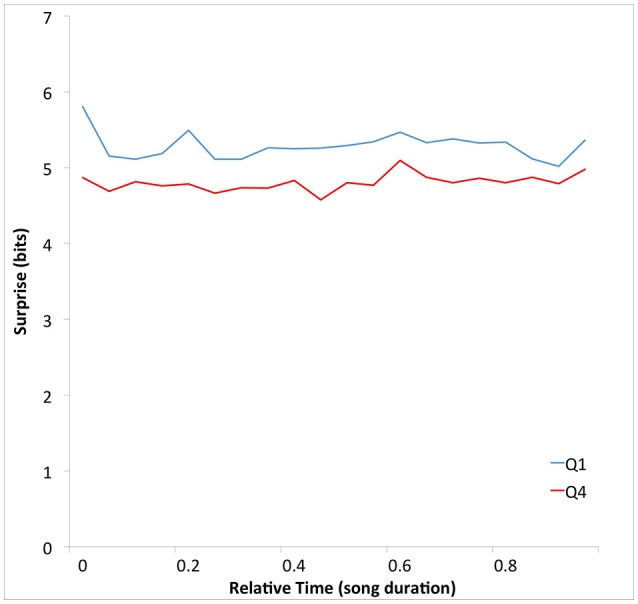
**Mean surprise as a function of time throughout the duration of songs for Q1 and Q4**. The abscissas were normalized by the total duration of the songs. The mean surprise is higher in Q1 than in Q4 songs throughout their durations. Again, the superiority of Q1 songs is consistent with the Absolute-Surprise Hypothesis.

Having found evidence supporting the Absolute-Surprise Hypothesis, we performed an additional exploratory analysis to see if any absolute-surprise effect might be localized at a specific relative temporal location in the songs. We plotted surprise as a function of time to determine if this was the case. To obtain this plot, we calculated average surprise over 12 non-overlapping relative-time bins. For this purpose, we first portioned each song into 12 equal portions. The bins were (B_1_, B_2_, …, B_20_) = (0–T/20, T/20–2T/20, …, 19T/20–T), where T was the duration of the song. Then for each bin B_*j*_, we calculated average surprise with Equation (2), setting κ to “B_j_.” The results of this calculation appear in Figure 3.

Figure 3 shows that the average surprise is higher in Q1 than in Q4 songs throughout their durations. As in Figure 2, the surprise difference is small (0.47 bits). However, the separation between these curves is highly consistent, for all 20 time-bins. This seems to contradict the notion that there is an absolute-surprise effect that is localized at a specific relative temporal location in the songs.

### Contribution of specific chord types to absolute-surprise effect

**Figure 4:**
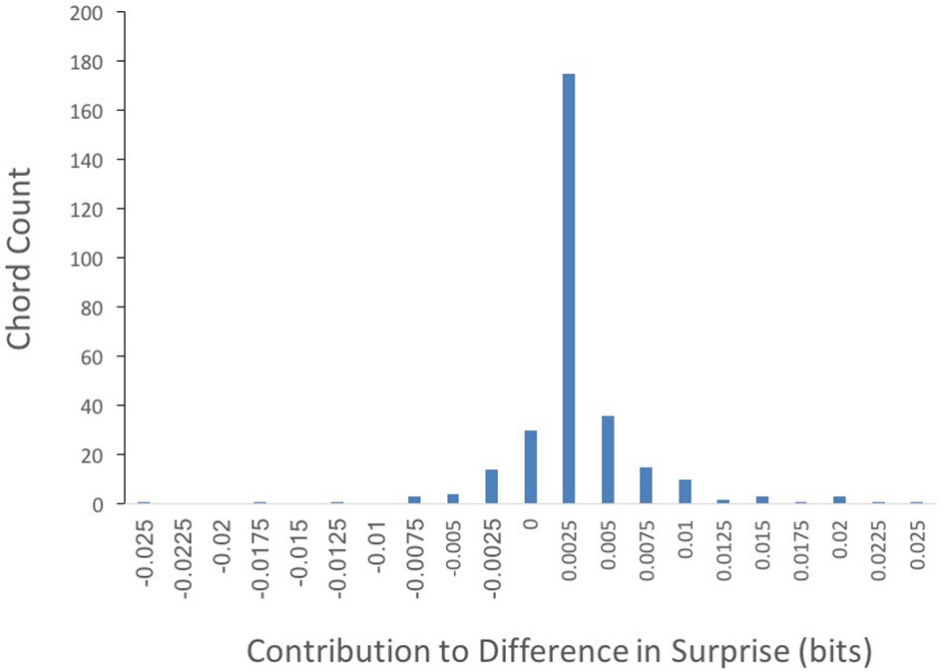
**Frequency of Q1 chords by their contribution to the surprise difference between Q1 and Q4 (Equation 3, with κ set to “whole song”)**. The majority of chord types contribute to the superiority in surprise of Q1 songs.

Could the difference in absolute surprise between Q1 and Q4 songs be accounted for by a small group of highly surprising chords? To answer this question, we wanted to know which chords within Q1 might be accounting for the difference in surprise between Q1 and Q4 songs. We calculated this difference as in Equation (4), settingκto “whole song.” The results of this calculation can be seen in Figure 4.

Out of 302 different chords featured in Q1 songs, the contribution to the difference in surprise between Q1 and Q4 was positive in 247 of them. In contrast, only 55 chords exhibited non-positive contributions. Thus, most chord types featured in Q1 songs contribute to their superiority in surprise. This result is inconsistent with a small group of highly surprising chords driving the difference in absolute surprise between Q1 and Q4 songs.

**Figure 5:**
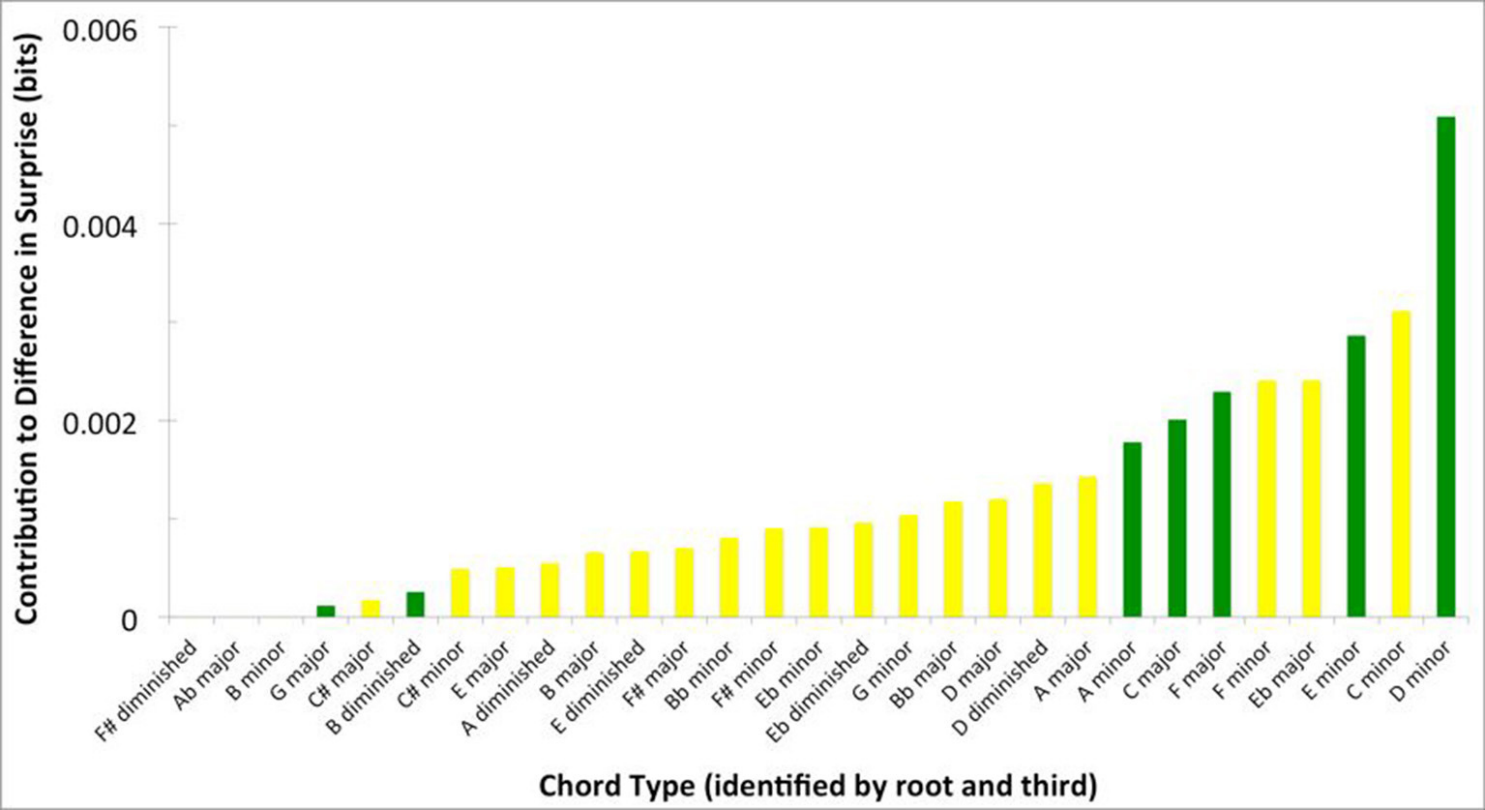
**Contribution of chords featured in Q1 songs and grouped by “root and third” to the surprise difference between Q1 and Q4**. Chords are organized from smallest to largest contribution. Green bars reflect diatonic chords, while yellow bars reflect chromatic chords. Note that diatonic chords contribute significantly to the difference in surprise between Q1 and Q4.

Figure 5 presents information, more specific to tonality, about the chords contributing to the difference in surprise between Q1 and Q4 songs. For this figure, we calculated the mean contribution of surprise (Equation 4, with κ set to “whole song”) of chord labels simplified by common tonality, that is, sharing roots and thirds. By combining chords in this manner, we can identify these simplified chord labels. Moreover, we can easily distinguish diatonic chords (green in the figure) and those with a root or third outside the key (yellow). Many of the chords that appear to contribute significantly to the difference in surprise between Q1 and Q4 are diatonic chords. Therefore, the contribution to this difference by some of the chords may be due to the prevalence of their extensions, rather than tonality based on their root or third notes.

### The effect of contrastive surprise

**Figure 6:**
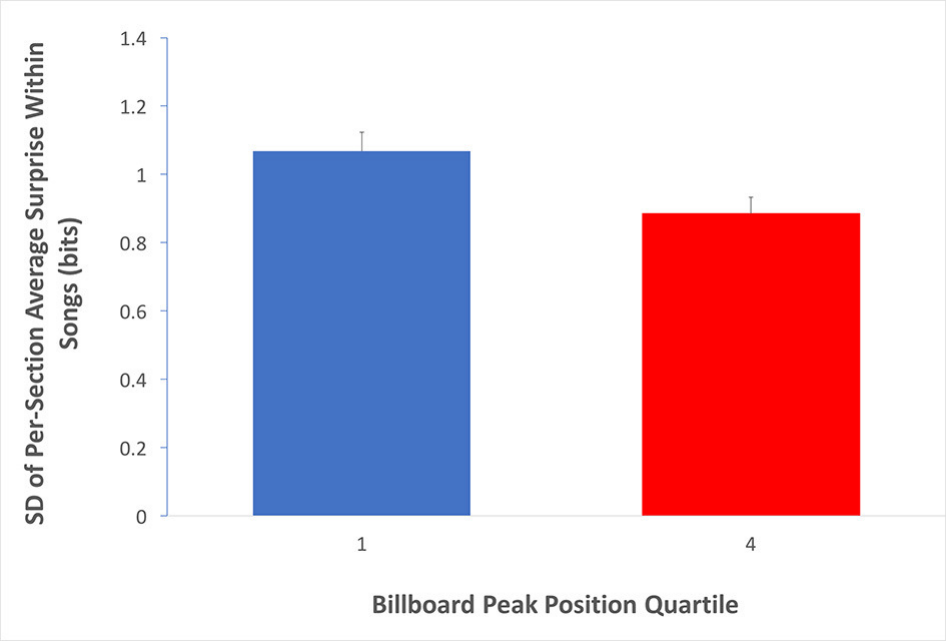
**Evidence for the Contrastive-Surprise Hypothesis**. Average per-quartile standard deviation across sections within songs (Equation 6, with κ set to “whole song”). Error bars are standard errors. The average surprise of sections within Q1 songs varies more than that of sections within Q4 songs. This finding is consistent with the Contrastive-Surprise Hypothesis.

So far, our data are supportive of the Absolute-Surprise Hypothesis. However, the Contrastive-Surprise Hypothesis has not been ruled out by these data. A natural place where contrastive surprise may appear is in the transition between sections. The form of popular music during this time period is characterized by alternating sections of contrasting harmonic structure, typically verses, choruses and bridges (Stephenson, 2002). Hence, if transitions across sections contributed to music preference, then we would expect a higher degree to which the sections within songs varied in their average surprise in Q1 than in Q4. Fortunately, labels for various song sections have been provided within the transcriptions of the McGill Billboard Project corpus, making such an analysis possible. We thus proceeded to measure the standard deviation of average surprise values for the different sections within each song (Equation 6, with κ set to “whole song”). Then, we compared these standard deviation values for Q1 to the corresponding values for Q4. If the Contrastive-Surprise Hypothesis were right, we would have expected to see higher mean standard deviation for average section surprises in Q1 than Q4 songs. The results of this analysis appear in Figure 6.

Standard deviations of average surprise for sections within individual songs were significantly higher for Q1 than for Q4 (Q1 mean = 1.07 bits, Q4 mean = 0.89 bits; two-tailed *t*-test; *t* = 2.47; *df* = 299; *p* < 0.05; Cohen's *d* = 0.280). Therefore, the Contrastive-surprise Hypothesis is supported by our data, providing evidence that the juxtaposition of high-surprise sections and low-surprise sections may indeed drive music preference upward.

### Localization of contrastive-surprise effect

**Figure 7:**
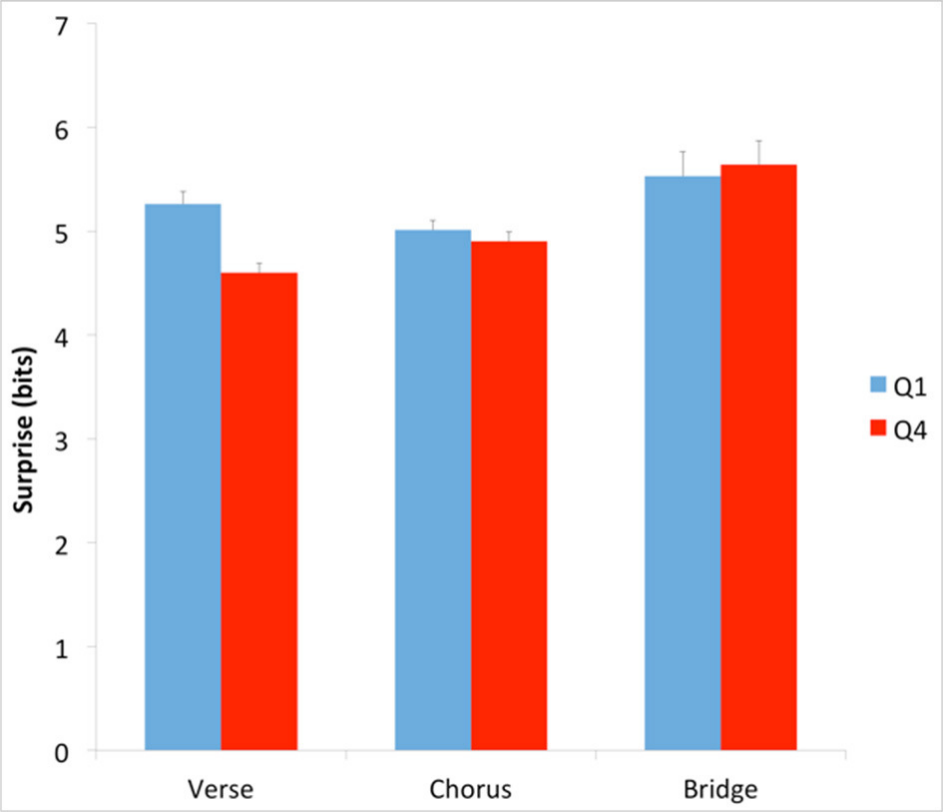
**Average surprise across Q1 and Q4 verses, choruses, and bridges**. Error bars are standard errors. Verses, but not choruses and bridges, account for the difference in surprise between Q1 and Q4.

Having found evidence supporting the Contrastive-Surprise Hypothesis, we again performed an additional analysis, as was done with the absolute-surprise effect. This time, the goal of our analysis was to see if any contrastive-surprise effect was localized across specific types of sections in the songs. We first asked whether the difference in absolute surprise between Q1 and Q4 songs extended to different sections within the songs. The three most prevalent labels throughout the corpus dataset are “verse,” “chorus,” and “bridge.” We used these labels to examine surprise in these types of sections within Q1 and Q4 songs. To do so, we calculated average surprise with Equation (2), setting κ to “verse,” “chorus,” or “bridge.” The results of this calculation appear in Figure 7.

Our results showed that verses, but not choruses or bridges, accounted for much of the difference in surprise between Q1 and Q4 (Figure 7). The difference was statistically significant for verses (Q1 mean = 5.26 bits, Q4 mean = 4.60 bits; two-tailed *t*-test; *t* = 4.31; *df* = 400; *p* < 0.001; Cohen's *d* = 0.325). There was no such difference between Q1 and Q4 in choruses or bridges. We performed an additional analysis of mean surprise of songs in Q1 and Q4 after removing all verses from the songs. This analysis used Equation (2), setting κ to “songs with verses removed.” The result showed that without verses, Q1 and Q4 songs are no longer statistically significantly different.

**Figure 8:**
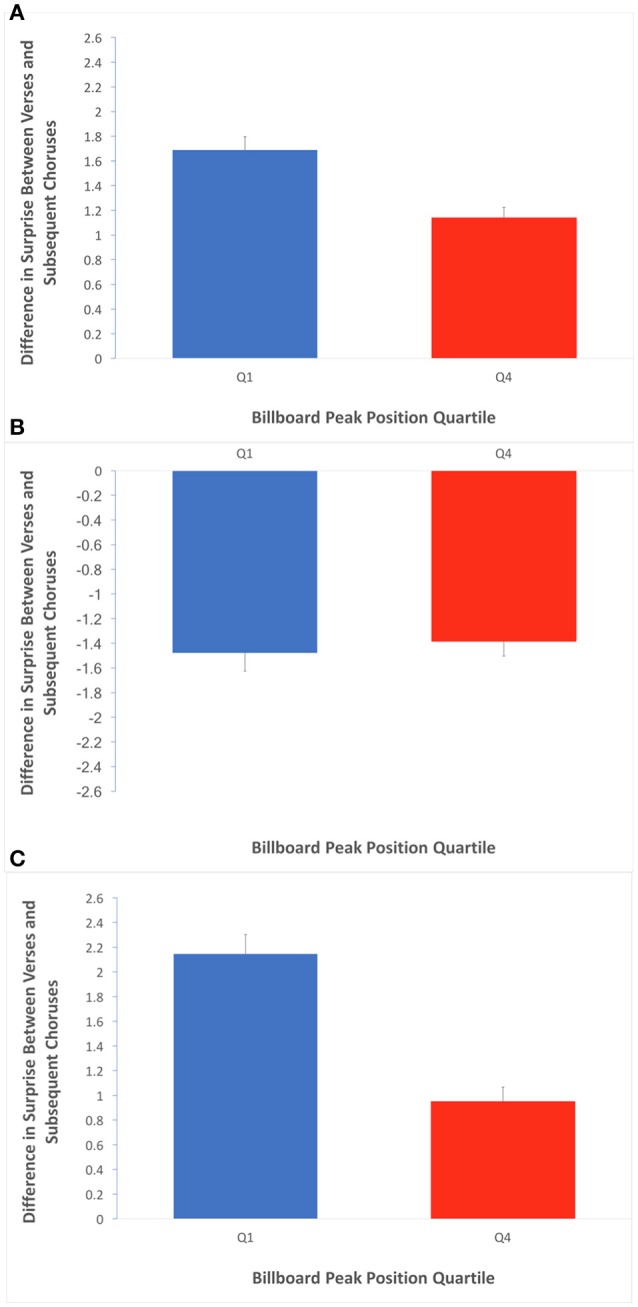
**Average difference in surprise between verse preceding a chorus and their subsequent choruses**. Error bars are standard errors. **(A)** Average of absolute values of differences in surprise over the verse/chorus transitions, for Q1 and Q4. The greater value in Q1 is consistent with an effect of contrastive surprise localized in the verse/chorus pairs. **(B)** Average of negative subset of differences when chorus surprise is subtracted from verse surprise. The lack of a quartile effect here suggests any contrastive-surprise effect between verses and choruses is not bidirectional. **(C)** Average of positive subset of differences when chorus surprise is subtracted from verse surprise. The greater value in Q1 is consistent with a contrastive-surprise effect that involves a drop in surprise from verse to subsequent chorus, specifically.

These results raised the possibility that the verses occurring before a chorus would have relatively high surprise, creating a tension in the song. The role of the subsequent chorus and its lower surprise would then be to release this tension. We then tested whether verse-to-chorus transitions might support the Contrastive-Surprise Hypothesis (Equations 7–9, with κ set to “whole song”). There were 209 and 193 such transitions in Q1 and Q4 respectively. We first examined the results in terms of any surprise difference at all (Equation 7). Afterwards, we examined negative differences when subtracting the surprise of chorus from that of the preceding verse (Equation 8) and equivalent positive differences (Equation 9). The results of these tests appear in Figure 8.

Figure 8A shows a significant effect of quartile when measuring overall difference in surprise (regardless of sign) across transitions from verses preceding a chorus to subsequent choruses (Q1 mean = 1.70 bits, Q4 mean = 1.14 bits; two-tailed *t*-test; *t* = 3.98; *df* = 400; *p* < 0.001; Cohen's *d* = 0.423). This result suggests that there is a contrastive-surprise effect that is accounted for by the relationship between verses and choruses. Furthermore, while Figure 8B shows that the effect is not significant for the subset of negative differences when the surprise of choruses are subtracted from their preceding choruses, Figure 8C shows it is significant for positive such differences (Q1 mean = 2.15 bits, Q4 mean = 0.95 bits; two-tailed *t*-test; *t* = 5.76; *df* = 400; *p* < 0.001; Cohen's *d* = 0.960). The results in Figure 8 suggest that the effect of contrastive surprise between verses and choruses is largely accounted for by decreases in average surprise from verses to subsequent choruses.

### The time course of contrastive surprise

**Figure 9:**
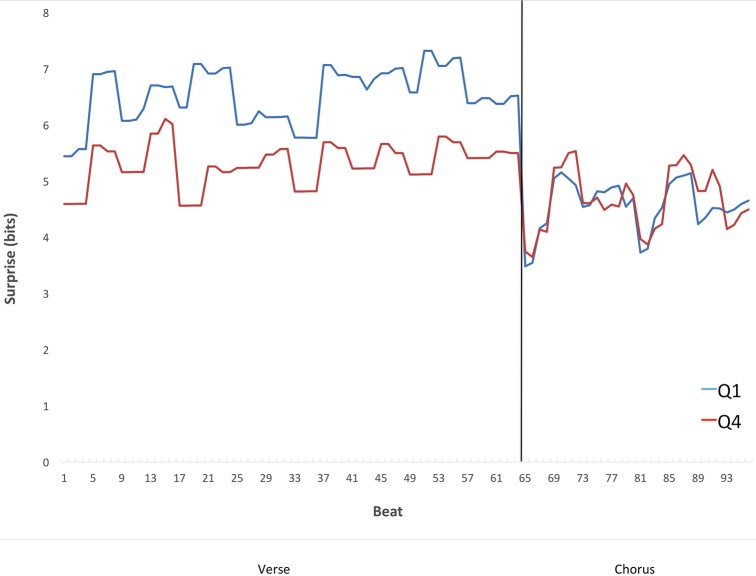
**Mean surprise as a function of time in the transition from verses preceding a chorus to the subsequent choruses from Q1 and Q4 songs, within the subset of verses that are 16 bars long, and within the subset of choruses that are 8 bars long**. The per-section average surprise falls from the verses to the choruses in Q1 but not Q4. The fall in Q1 songs is consistent with the Contrastive-Surprise Hypothesis.

Figure 8 suggests that the contrastive-surprise transitions from verses to choruses may contribute to the difference in preference for Q1 vs. Q4 songs. However, Figure 8 does not address the time course of these transitions. In particular, we wanted to know whether the increased surprise in Q1 verses was localized at a specific temporal area of the verse. For example, could surprise in verses increase just before the transition, making contrastive surprise even stronger? We chose to look at the time course of these transitions in 16-measure verses preceding a chorus and the following 8-measure choruses. These durations are the most prevalent of each type of section in the corpus. From these, we selected only the sections in 4/4 meter, also the most prevalent type in the corpus. The idea was to display a representative time course of the effect, with sections sufficiently homogeneous in their timing to provide useful information. We averaged the surprise for each beat of the verses (64 beats) and each beat of the choruses (32 beats). To perform this average, we used Equation (2), with κ conditioning the analysis to the types of verses and choruses as specified above and set to “each beat.” The results appear in Figure 9.

The results in Figure 9 show that the surprise in the verses is not rising just before the choruses to increase contrastive surprise. If anything, surprise appears to fall in the verses before the transition to the choruses in Q1 but not Q4. Statistical analysis shows that the time course of surprise in the 16-bar 4/4 pre-chorus verses tends to be a convex arch, i.e., featuring a rise in surprise from the first measure to the average of the middle 14 measures, and then a fall in surprise by the final measure. Convex arches were significantly more common than concave in both Q1 (χ^2^ = 13.89, *df* = 1, *p* < 0.001) and Q4 (χ^2^ = 4.60, *df* = 1, *p* < 0.05). Interestingly, this tendency toward convexity is similar to findings elsewhere (Huron, 1996) with measures of pitch within melodic phrases, including the apparent dip halfway through the arch. Therefore, the preferred time course of surprise may be continuous rather than discontinuous at the verse-chorus transition.

### Genre as a possible confound

The two main findings reported above are an effect of quartile on per-song average surprise and an effect of quartile on standard deviation of per-section average surprise within songs. We have presented these findings as support for the Absolute-Surprise Hypothesis and the Contrastive-Surprise Hypothesis, respectively. It is possible, however, that in each of these two cases there might be a third variable that is somehow driving the relationship between the two factors that are reported. Such a variable would, in theory, have a significant effect on measures of both harmonic surprise and preference.

Genre is one such potentially confounding variable. If a particular genre were more (or less) preferred than another, and there were an unrelated but significant difference between these genres in either per-song average surprise or standard deviation of per-section average surprise within songs, then genre itself could be the factor driving the effects reported above. This would call into question whether the findings support either the Absolute-Surprise Hypothesis or the Contrastive-Surprise Hypothesis. Furthermore, if genre were a confounding variable for either quartile effect (average per-song surprise or average standard deviation of per-section surprise within songs), that effect would not be likely to be detected within the various genres in isolation.

To investigate whether genre was a confounding factor, we labeled the songs of Q1 and Q4 by genre. Using labels obtained from the website All music (http://www.allmusic.com), we classified the songs under three genre labels. We determined 174 songs to be “Rock,” 89 songs to be “R&B,” and 35 songs to be “Country” (three songs were classified under the genre label of “Musical Theater,” and were not included in this analysis). We then performed two 3 (genre) by 2 (quartile) analyses of variance (ANOVAs)—one for each effect. The first was a Factorial ANOVA comparing the main effects of quartile and genre, and the interaction effect between genre and quartile, on average per-song surprise. The first ANOVA yielded no significant interaction effect. It showed that for average per-song surprise, there was a small, but statistically significant main effect of quartile (*F* = 8.14; *df* = 1, 292; *p* < 0.01; partial eta squared = 0.027), suggesting the higher surprise in Q1 songs is not isolated to a single genre. There was also a significant effect of genre (*F* = 12; *df* = 2, 292; *p* < 0.001; partial eta squared = 0.081). The second test, of standard deviation of per-section average surprise per song, did not yield a significant interaction effect or main effect of quartile. The lack of main effect of quartile could be due to the small sample size of Country songs (there were only seven Country songs in Q1). We subsequently performed a 2 (genre) by 2 (quartile) analysis of variance, with Rock and R&B songs only. This test showed that for standard deviation of per-song average surprise per song, there was no interaction effect, and a small, but statistically significant main effect of quartile with Rock and R&B songs only (*F* = 4.11; *df* = 1, 259; *p* < 0.05; partial eta squared = 0.016). This test also yielded a significant main effect of genre (*F* = 6.96; *df* = 1, 259; *p* < 0.01; partial eta squared = 0.026). The significant main effects of quartile suggest that although the genres seem to differ in their harmonic surprise, songs from different genres contribute to the observed quartile effects supporting both hypotheses. These findings are inconsistent with the notion that genre is a confounding variable that could entirely account for either effect.

## Discussion

### Findings and their support for the proposed hypotheses

In this paper, we set out to test two hypotheses for how harmonic surprise might contribute to preference in popular music. We call the first the Absolute-Surprise Hypothesis. It states that moderate increases in the absolute level of surprise directly evoke pleasurable emotions, thus driving preference upward. In turn, we termed the second the Contrastive-Surprise Hypothesis. It states that overall preference is driven by passages with moderately high surprise, thus evoking unpleasant emotions of “tension,” followed by passages with low surprise, evoking a pleasurable “release” from these emotions. We tested these hypotheses by comparing harmonic surprise and its time course in songs within the top and bottom quartiles of peak Billboard chart position from a representative corpus. Both hypotheses are supported by our data (Figures 2, 6). Furthermore, the observed surprise effects did not depend on a small number of special chords (Figure 4). Although the findings support both hypotheses, it is possible that some of the observed effects could be artifactual, a possibility which we will now explore.

Under the Absolute-Surprise Hypothesis, an absolute-surprise effect is either dominating or entirely driving the relationship between harmonic surprise and preference in popular music. Under this interpretation, the high surprise of verses alone within Q1 could be providing emotional stimulation, leading to reward, within listeners. The listeners are responding to this reward, originating in the high-surprise verses, when developing preference. It is possible that verses, which we found to be driving the quartile effect of surprise, are the most advantageous sections for this tactic within music composition. Thus, under the Absolute-Surprise Hypothesis, the appearance of structures featuring contrastive surprise (Figures 7–9) is simply an artifact, not a feature. If the difference between Q1 and Q4 songs is that the former has verses with more surprise, then in Q1 the verses preceding a chorus also have more surprise. Thus, the transitions from these verses to these choruses would feature more surprise contrast as a side effect of the heightened surprise in verses. Rather, the Absolute-Surprise Hypothesis proposes, the unexpected stimulus itself is responsible for evoking the pleasurable response leading to preference. There is some precedent for this conclusion outside the domain of music cognition. In a study in the visual domain, unexpected stimuli were shown to evoke a dopamine response (Kakade and Dayan, 2002). In this study, the reward response was attributed to an evolutionarily adaptive attraction to novelty.

Under the Contrastive-Surprise Hypothesis, a contrastive-surprise effect is either dominating or entirely driving the relationship between harmonic surprise and music preference. Under this interpretation, it is possible that unpleasant emotions evoked through the high-surprise verses preceding a chorus in Q1 are “resolved” once the low-surprise choruses set in. This resolution could lead to reward, and thus preference. Under the Contrastive-Surprise Hypothesis, the relatively higher values of surprise in Q1 songs (Figure 3) are an artifact. Under this hypothesis, levels had to be high to improve preference, because otherwise a release of tension through the lowering of surprise would not be possible. Moreover, the flatness of the temporal behavior of surprise would be artifactual, too. This flatness, despite the contrastive behavior observed in Figure 9, might be caused by the scattering of choruses and verses throughout the duration of different songs. This scattering might cause the associated surprise values in different bins to average to roughly the same values. Under the Contrastive-Surprise Hypothesis, the contrast between an unpleasant stimulus and the subsequent subsiding of that stimulus is responsible for the pleasurable response driving preference. There is a precedent for the proposed mechanism of the Contrastive-Surprise Hypothesis outside music cognition as well. Participants in a previous study were shown to have increased endogenous opioid response after exposure to cognitive stressors (Bandura et al., 1988). In that study, the reward response was attributed to the juxtaposition of the presence and removal of the cognitive stressors.

An alternative at least in part compatible with our data is that a complex combination of absolute and contrastive forms of surprise is what drives preference. We call this potential alternative the *Hybrid-Surprise Hypothesis*. Both Meyer's (1956) and Huron's (2006) theories suggest that they may agree with this hypothesis, because both discuss absolute and contrastive mechanisms. As argued above, both absolute and contrastive forms of surprise can be advantageous for the observer. Perhaps, each of these forms is advantageous under different conditions, requiring the brain to perform a calculation that is more intricate to decide whether to like the stimulus. For example, the Absolute-Surprise Hypothesis might be specifically well-suited to familiar music. It is possible, according to Huron's model of anticipatory response to music (Huron, 2006), that the positive appraisal response to some unexpected aspects of stimuli occurs almost instantaneously during familiar pieces of music, immediately following the negative reaction response. Thus, enjoyment could occur without a subsequent release of tension in the music itself. This might explain the results of Salimpoor et al. (2011), where coordinated fMRI and PET results seem to indicate dopamine release contemporaneous with harmonically surprising events within stimuli known to evoke “chills.” The “chills”-evoking stimuli in that study were highly familiar to the participants. Perhaps with an unfamiliar piece, enjoyment of music with a high-surprise section tends to require a lower-surprise section to prompt the positive appraisal response. With higher familiarity, the positive appraisal could be provided by conscious comfort with the piece itself. Thus, familiarity might be one factor mediating a Hybrid-Surprise Hypothesis.

### Limitations of our tests of the hypotheses

The reason that our tests could not distinguish between the two proposed hypotheses is, at least in part, methodological. Our goal was to perform these tests with naturalistic music, that is, avoiding the over-simplifications of structure that sometimes arise in laboratory tests. What we gained by performing our tests in this way is evidence consistent with hypotheses about how harmonic surprise may matter in the real world. However, what we lost was the ability to carefully control various forms of surprise. In this section, we discuss the limitations of our methods.

Our tests were the product of a limited investigation into a specific aspect of popular music, namely harmonic surprise. There are several other aspects of music that could contribute to preference, or even interact with the aspects studied here. For example, our analyses focused on preference based on zeroth-order surprise calculated from the distribution of chords. They did not investigate harmonic surprise at any higher-order level of organization, as other “n-gram” music corpus studies might (Patel and Mundur, 2005). Curiously, informal experiments replicating our analyses at the level of first-order and second-order transitions did not produce significant differences between the quartiles. This could be due to the expanding demands of dimensionality required to demonstrate effects at higher-order levels of organization. It also could be because such effects do not exist, or because they are more complex at higher-order transition levels. In addition, our investigation was limited to the statistical prevalence of chords. Additionally, this study of surprise in chords does not take into account any possible influences on preference that do not involve harmony. It is possible that there are also aspects having to do with the timing of the chords that contribute to music preference. These aspects could contribute to preference either independently or in interaction with the effects reported here. There have been claims of such contributions to music preference (Rohrmeier and Koelsch, 2012).

We also chose to limit our investigation into a specific subset of popular music, namely, the songs that reached the Billboard *Hot 100* between the years of 1958 and 1991. It could be argued that this subset does not represent the full scope of Western popular music. One possible consequence of this choice is that since we chose music that, by definition, met a certain threshold of commercial success, our analysis might be missing the ends of a possible “inverted-U” shape in the effects we observed. Such an effect, first proposed by Berlyne et al. (1966), would involve a drop in either one of the two effects we proposed, at the extreme ends. This is contrary to the rather linear effects we proposed in our hypotheses. It is possible that this “inverted-U” shape is artificially truncated due to the constraints we placed on the study by including only commercially successful songs.

Another limitation of this study is its methodology for measuring preference. Measuring preference is more straightforward in an experimental setting. It may be argued that our chosen measure for preference, namely peak Billboard chart position, is influenced by many factors beyond simple preference. The most significant of these factors, radio airplay, might have been subject to commercial pressures and other forces independent of any listener's personal preference. Unlike with genre, however, it is unlikely that radio airplay is also correlated with harmonic surprise in any significant way. This is also the case with other factors that might influence music preference that do not involve the structure of music, such as artist attractiveness, artist gender, etc., Hence, we feel that we diminished statistically the influence of such potential confounds.

Finally, yet another limitation is that our findings are correlational. We found evidence of correlation between harmonic surprise (both absolute and contrastive) in a representative corpus of popular music and peak ratings on the Billboard Hot 100 chart. We cannot conclude that this evidence shows that increased surprise (of one kind or the other) leads to higher preference in popular music. Future behavioral studies must be conducted to test whether a causal link between harmonic surprise and preference exists. Together with neuroimaging studies, these tests might help to tease apart the various hypotheses that we have proposed, and shed further light on the neural correlates of reward linked to specific aspects of music.

## Author contributions

SM was involved in the design of the project, developing, and using programming scripts for data analysis, interpreting results, and writing and revising the manuscript. DR was involved in the design of the project and revising the manuscript. NG was involved in the design of the project, developing the mathematical equations, interpreting the results, and revising and approving the manuscript for publication.

## Funding

Funding was provided to SM by a Graduate Research Fellowship from the National Science Foundation.

### Conflict of interest statement

The authors declare that the research was conducted in the absence of any commercial or financial relationships that could be construed as a potential conflict of interest.
